# CPORT: A Consensus Interface Predictor and Its Performance in Prediction-Driven Docking with HADDOCK

**DOI:** 10.1371/journal.pone.0017695

**Published:** 2011-03-25

**Authors:** Sjoerd J. de Vries, Alexandre M. J. J. Bonvin

**Affiliations:** Faculty of Science, Bijvoet Center for Biomolecular Research, Utrecht University, Utrecht, The Netherlands; Leeds Institute of Molecular Medicine, United Kingdom

## Abstract

**Background:**

Macromolecular complexes are the molecular machines of the cell. Knowledge at the atomic level is essential to understand and influence their function. However, their number is huge and a significant fraction is extremely difficult to study using classical structural methods such as NMR and X-ray crystallography. Therefore, the importance of large-scale computational approaches in structural biology is evident. This study combines two of these computational approaches, interface prediction and docking, to obtain atomic-level structures of protein-protein complexes, starting from their unbound components.

**Methodology/Principal Findings:**

Here we combine six interface prediction web servers into a consensus method called CPORT (Consensus Prediction Of interface Residues in Transient complexes). We show that CPORT gives more stable and reliable predictions than each of the individual predictors on its own. A protocol was developed to integrate CPORT predictions into our data-driven docking program HADDOCK. For cases where experimental information is limited, this prediction-driven docking protocol presents an alternative to *ab initio* docking, the docking of complexes without the use of any information. Prediction-driven docking was performed on a large and diverse set of protein-protein complexes in a blind manner. Our results indicate that the performance of the HADDOCK-CPORT combination is competitive with ZDOCK-ZRANK, a state-of-the-art *ab initio* docking/scoring combination. Finally, the original interface predictions could be further improved by interface post-prediction (contact analysis of the docking solutions).

**Conclusions/Significance:**

The current study shows that blind, prediction-driven docking using CPORT and HADDOCK is competitive with *ab initio* docking methods. This is encouraging since prediction-driven docking represents the absolute bottom line for data-driven docking: any additional biological knowledge will greatly improve the results obtained by prediction-driven docking alone. Finally, the fact that original interface predictions could be further improved by interface post-prediction suggests that prediction-driven docking has not yet been pushed to the limit. A web server for CPORT is freely available at http://haddock.chem.uu.nl/services/CPORT.

## Introduction

Macromolecular complexes are the molecular machines of the cell. In order to fully understand how the various units work together to fulfill their tasks, structural knowledge at the atomic level is required. An atomic-resolution structure is also an important first step in rational drug design and other efforts to influence the function of macromolecular complexes, which is of high medical relevance.

The classical methods to obtain atomic-resolution structures are X-ray crystallography and Nuclear Magnetic Resonance (NMR). In recent years, tens of thousands of single protein structures have been solved using these methods, as well as an increasing number of complexes. However, the number of expected macromolecular complexes will exceed the number of proteins in a proteome by at least one order of magnitude [1]. Since complexes are often weak, dynamic and/or very large, a significant fraction of these will be extremely difficult to study using any experimental method. Therefore, the importance of large-scale computational approaches in structural biology is evident [2].

This study combines two of these computational approaches, interface prediction and docking. Interface prediction aims, by computational means, to identify the residues on the protein surface that interact with another protein or biomolecule. Docking takes this one step further by predicting the three-dimensional structure of a protein complex, starting from the free, unbound structures of its constituents.

Interface prediction is based on the extraction and combination of distinguishing features from protein sequences and structures. Genomic and structural genomic initiatives, combined with advances in computer technology, have allowed protein interfaces to be analyzed and predicted today in a far more systematic way than what was possible in the past. While older methods could only be tested on a case-by-case basis or on a small set of similar complexes, large-scale statistical analysis and validation on non-redundant benchmarks has become the norm. Therefore, interface prediction is a field that is rapidly developing. For two recent reviews on interface prediction, see Zhou and Qin [3] and de Vries and Bonvin [4].

Similar developments have benefited the docking field as well. The protein-protein docking benchmark 2.0 [5] represents a large and diverse set of complexes and forms a testing ground for the development of new methods. In addition, to monitor the performance of current docking methods, CAPRI (Critical Assessment of Predicted Interactions), a community-wide blind docking experiment, has been established (http://capri.ebi.ac.uk). In this experiment, participants are asked to predict by docking a recently solved protein-protein complex a few weeks prior to its publication. The large majority of the recent targets had to be predicted using only unbound structures or even homology models. Also, recent targets have a higher representation of biologically interesting signal transduction complexes, which are known to be difficult to dock. Despite these challenges, successful predictions were made for several targets that were considered beyond the limits of docking methodology a few years ago.

In general, docking methods can be divided into *ab initio* and data-driven docking methods. Data-driven docking means that experimental information is used directly during the docking process, so that the only possible solutions are those that agree with experiment. The most widely used data-driven docking method, HADDOCK [6], [7], [8], was developed in our group. HADDOCK is currently the most-cited docking method in the world: it is widely used for structure calculation of protein complexes using NMR data, and more than 70 experimentally-determined structures have been solved and deposited in the PDB. HADDOCK has been applied to a variety of problems including protein-protein, protein-nucleic acids and protein-small molecule complexes, in combination with a wide range of experimental data, ranging from NMR, mass spectrometry to mutagenesis data (for an overview, see [9], [10]).

However, the sophisticated use of experimental data in HADDOCK, which is its strength, also imposes a limitation. *Ab initio* docking in HADDOCK, while possible, performs poorly compared to state-of-the-art docking methods, limiting the effective use of HADDOCK to cases where sufficient experimental information is available. In principle, interface predictions can be used to remove this limitation and there have been previous attempts in this direction [3], [11]. However, until now, no interface prediction method has been reliable enough to be combined with docking and then applied to a wide range of protein-protein complexes.

The aim of this work is to derive from interface predictions a set of optimal restraints for data-driven docking using HADDOCK. This can then serve as a starting point for docking cases where experimental information is limited. To achieve this aim, six interface prediction web servers were combined in a consensus method called CPORT (Consensus Prediction Of interface Residues in Transient complexes). CPORT predictions were used to dock the full protein-protein benchmark, excluding only antibody-antigens and multimer complexes, using HADDOCK. CPORT predictions were shown to be more reliable than the best individual predictor, PINUP, and resulted in at least an acceptable docking solution in the top 400 for the majority of the complexes.

## Results

The aim of this work is to derive from interface predictions a set of optimal restraints for data-driven docking. Interface predictors often disagree strongly with each other; in most cases, at least one predictor will be correct but it is not possible to tell which one [4]. One way to deal with this problem is meta-prediction: by parametric combination of interface prediction scores, a new score can be computed that is more specific than any of the individual scores. We have previously made such a combination of WHISCY and ProMate [11], and this approach has also been adopted by Qin and Zhou [12] and more recently by Huang and Schroeder [13].

However, the maximization of overall specificity is not the best approach when interface predictions are meant to drive the docking in HADDOCK. We found that HADDOCK is consistently able to deal with fuzzy data, i.e. data where correct interface predictions are mixed with wrong ones. It is, however, essential to cover at least some part of the interface, and this must be the case for both partners, because correct solutions will not be sampled otherwise. Therefore, we opted for a consensus strategy, selecting residues that are predicted by one or another predictor, rather than combining them into a new score. We also chose to deliberately overpredict the interface, relying on the HADDOCK scoring function to discriminate between correct and incorrect docking solutions. Together, this minimizes the risk that an interface prediction is entirely wrong, and increases the chance of success in prediction-driven docking.

We must emphasize that the current work is specifically aimed at the use of interface predictions in data-driven docking with HADDOCK. In the literature, many different test statistics have been used to evaluate interface predictions, including specificity, sensitivity, Matthews correlation and AUC (Area Under the Curve) (see [4] for a review). The assessment of interface predictions is further complicated by the fact that proteins often have alternative interfaces with different partners, and that this should be properly accounted for in the computation of “true” statistics. In a docking context, however, only the interface with the docking partner is relevant, and only test statistics with regard to this interface could have any relationship to the outcome of the docking. Finally, our emphasis on achieving good sensitivity and minimizing the chance of entirely wrong predictions comes from our experience with HADDOCK and may not be true in a different docking context.

### Collecting interface prediction data

In order to develop a consensus prediction method and to test it in docking, the protein-protein docking benchmark 2.0 [5] was chosen. This is a non-redundant benchmark of complexes of which both bound and unbound structures are available. We took all complexes in the “enzyme” and “other” categories, since antibody-antigens are not suitable for interface prediction [3], [4], [14], resulting in a dataset of 59 complexes. The unbound forms of these complexes were sent to each of the six web servers (WHISCY, PIER, ProMate, cons-PPISP, SPPIDER, and PINUP) and the prediction scores were extracted. We found that it was better to use the rank of the prediction score, rather than its absolute value, except for SPPIDER. A detailed analysis is given in figure S1.

An important issue is whether predictions should be made on bound or unbound forms. Since the goal is to use predictions in docking, and since bound docking has little biological relevance, we focused on the unbound forms for both prediction and docking.

In addition, we investigated the effect of switching from the unbound to the bound forms. Earlier literature suggested that interface predictors are insensitive to such small structural differences [4], [14]. However, we found considerable influence on several predictors: in particular, PIER and SPPIDER performed better on bound structures while PINUP performed better on the unbound forms (Figure S2).

While the complexes in the benchmark 2.0 are transient complexes, the large majority of complexes in the PDB are obligate: no unbound form is available for them because their chains are never separated *in vivo*. It has been shown that obligate interfaces differ considerably from transient interfaces in terms of size, shape, composition, contacts and conservation [15], [16]. This is another reason why only the protein-protein benchmark was used, and none of the hundreds of bound complexes available in the PDB.

The protein-protein benchmark is non-redundant in the sense that no complexes have homology for both partners. However, at the single protein level there is considerable redundancy, with proteins such as trypsin and its homologs represented several times with different partners and having somewhat or completely different residues in the interface. This means that independent cross-validation by partitioning is not possible. Thus, it is important to train a consensus predictor in a simple way, to prevent over-fitting of the data. This is also the reason why the set was not partitioned into training set and test set. However, in addition, an independent validation was performed on complexes not used in training, namely the new chains of the benchmark 3.0 [17].

### Consensus interface prediction

We assembled a training set of residues that was limited to those for which all predictors assigned a score. Prediction scores were converted to integer values, by simply taking the rank of the score within the protein chain (except for SPPIDER). Then, consensus prediction was done by deriving a number of optimal sets. Each set corresponds to a certain sensitivity and consists of the top X WHISCY predictions, the top Y ProMate predictions, the top Z PINUP predictions, etc. The goal is to find the optimal cutoff values for X,Y,Z,… for the given sensitivity.

We could have used regression to find the optimal values for each set, but this would have resulted in considerable risk of over-fitting. Instead, a simple, greedy algorithm was used (see Materials and Methods). Starting with an empty set (all six cutoffs X,Y,Z,… set to zero), new sets were generated by incrementing one of the cutoffs by 1. Therefore, there were only six different possibilities per set, with minimal chances of over-fitting.

Initial docking tests were then performed on six complexes using various degrees of interface overprediction (see Text S1). This resulted in the choice of an optimal cutoff with on average 50 predictions per chain. The resulting consensus interface predictor is called CPORT (Consensus Prediction Of interface Residues in Transient complexes).

The test set was then expanded into an evaluation set, with some additional chains and additional interface residues (see Materials and Methods). All six individual interface predictors, as well as CPORT, were evaluated on this set. For the six individual predictors, the top 30 residues were taken. Among them, we found PINUP to be the best-performing: for 47 of the 109 chains, PINUP was the best or tied for the best interface predictor. For PIER, ProMate, SPPIDER, WHISCY and cons-PPISP these numbers were 28, 21, 20, 18 and 15, respectively. PINUP was among the best three predictors, or tied for those, for 84 chains. For PIER, WHISCY, ProMate, SPPIDER and cons-PPISP these numbers were 71, 58, 56, 52 and 49, respectively. Therefore, it is clear that while PINUP is better than the other predictors, it is outperformed by at least one of those predictors in most cases, and that consensus interface prediction is in principle possible.

The performance of CPORT was evaluated and compared to PINUP (Table 1). The top 50 PINUP predictions were taken, so that on average the same number of predictions was made by PINUP and CPORT. It can be seen that CPORT predictions improve on PINUP, although the gain in performance is modest. The main improvement is that the number of complete failures, i.e. cases where all predictions are wrong, is halved. This meets an important goal, which is the reliable prediction of at least some part of the interface, because unless this requirement is met for both chains, data-driven docking will surely fail. However, the use of an interface predictor should not depend on solely this criterion; sensitivity and specificity should be considered as well. The percentage of proteins for which at least 40% sensitivity and/or specificity is achieved is a measure of the stability of the method. For these criteria, CPORT makes a modest improvement by two to five percentage points. The overall sensitivity and specificity over the predictions is nearly the same between the two predictors.

**Table 1:** Comparison between CPORT and PINUP predictions on the benchmark 2.0.

	All wrong	Sensitivity> = 40%	Specificity> = 40%	Sens & spec> = 40%	Overall sensitivity	Overall specificity
**CPORT**	2%	82%	24%	24%	53%	27%
**PINUP**	4%	80%	19%	19%	52%	27%

CPORT made on average 50 predictions per protein chain; shown is the comparison between CPORT and the top 50 PINUP predictions on the protein-protein docking benchmark 2.0 [5].

In addition, we compared CPORT to the other five interface predictors (Table S1) and to alternative meta-prediction schemes (Table S2). Also here, CPORT showed a more constant and reliable performance.

### Validation of CPORT on an independent test set

We also tested CPORT on all new complexes from version 3.0 of the benchmark [17], representing another 74 chains. This set of additional complexes was not used in the training of CPORT and differs in overall composition, containing fewer enzymes and more complexes that undergo large conformational changes. CPORT made only 45 predictions per chain on average for these complexes. Therefore, we compared CPORT with the top 45 predictions from PINUP (table 2). It can be seen that CPORT performs much better than PINUP, with all predictions wrong in only 3% of the cases, compared to 16% for PINUP. Sensitivity and specificity values are also much better for CPORT than for PINUP. The same was observed for alternative meta-prediction schemes (table S3).

**Table 2:** Comparison between CPORT and PINUP on the benchmark 3.0.

	All wrong	Sensitivity	Specificity	Sens & spec	Overall	Overall
		> = 40%	> = 40%	> = 40%	sensitivity	specificity
**CPORT**	3%	70%	26%	24%	48%	28%
**PINUP**	16%	54%	19%	19%	42%	25%

CPORT made on average 45 predictions per protein chain; shown is the comparison between CPORT and the top 45 PINUP predictions for the 37 new targets of the protein-protein docking benchmark 3.0 [17].

### Statistical significance of the improvements

For the benchmark 2.0, the observed improvements over PINUP were not large enough to be statistically significant (p = 0.235–0.432, Fisher exact test, one-tailed); the number of chains (109) is too small to detect differences of just a few percent. However, compared to all other individual predictors, CPORT was significantly better in sensitivity (p = 0.0001–0.02, Fisher exact test), and better in specificity than all methods except ProMate (p = 0.00064–0.041; CPORT vs ProMate, p = 0.119). This is not very surprising given the fact that CPORT outperforms all of these methods by at least ten percent.

For the benchmark 3.0, CPORT's improvements over PINUP were more substantial, and were statistically significant for the number of all wrong predictions (p = 0.0046), the number of predictions with sensitivity >40% (p = 0.031), the overall sensitivity (p = 0.00014) and the overall specificity (p = 0.0011).

### Correlation between sensitivity and the number of predictions

As a rule, in interface prediction, the more residues are predicted, the higher the sensitivity will be, usually at the cost of specificity. For example, taking all the WHISCY scores higher than zero as predicted interface results in a sensitivity that is well correlated with the number of predictions (r = 0.63). However, CPORT shows exactly the reverse: among the proteins in the benchmark, the sensitivity of the prediction is negatively correlated with the number of predicted interface residues (r = −0.35). This intriguing property is due to the fact that CPORT adds predictions from various interface predictors (rather than combining their scores), so that the predicted interface is small when the underlying interface predictors agree well, indicating a strong signal for the possible binding site. Disagreement between predictors, indicating multiple possible binding sites and/or a weak signal, results in a much larger number of predictions, increasing sensitivity for an interface that is otherwise elusive to interface prediction. In the context of data-driven docking, where fuzzyness in the data can be dealt with but sensitivity must be achieved at all cost, this property is very desirable.

### Docking results

CPORT predictions were used to drive the docking of the complexes in the protein-protein benchmark 2.0 using HADDOCK. A small training set of six complexes was used to find the optimal values for two docking parameters, namely the number of docking trials and the percentage of restraints to discard at random (see Text S1). After this, docking proceeded on all complexes from benchmark 2.0 except antibody-antigen complexes and multimers. For each complex, 10 000 structures were generated in the rigid body stage, of which the top 400 were refined. As a control, a docking run using HADDOCK in *ab initio* mode was performed for each complex. Finally, an alternative CPORT docking run was performed under slightly different conditions, resulting in better energies and a higher percentage of correct structures, but with considerable difficulty to discriminate correct from incorrect structures. Because of this, we decided from the beginning not to use the results of the alternative run in this study, but it is made available together with the other runs as decoy sets (see Text S1) for use in the development of better scoring functions.

**Figure 1:**
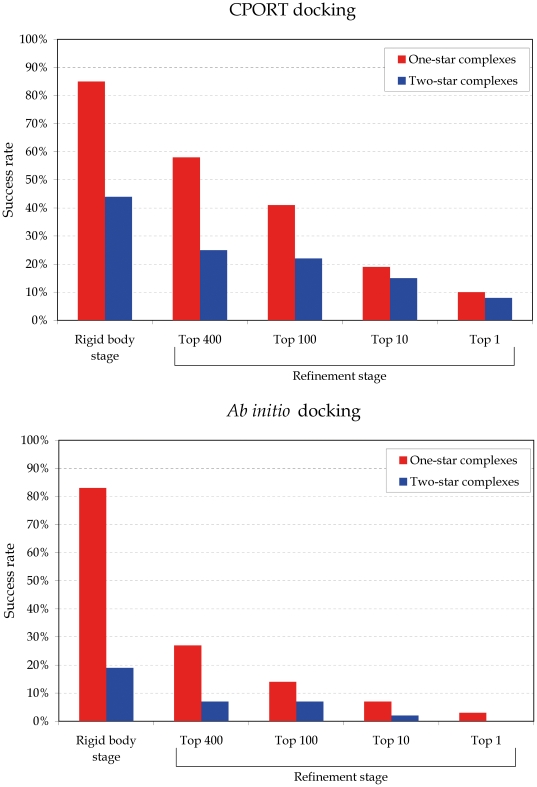
Docking results. Docking results for CPORT-driven docking using HADDOCK (top), compared to HADDOCK *ab initio* docking (bottom). The figure shows the percentage of cases for which at least one structure of that quality was generated during the rigid body stage (10 000 structures), and the top 400 (all refined structures), 100, 10 and 1 of the refinement stage. One-star and two-star criteria correspond to the CAPRI [18] definitions (see Methods). For the rigid body stage, the fnat criterion is not taken into account.

Figure 1 shows an evaluation of the docking results according to the CAPRI criteria [18] (see Materials and Methods), based the Root Mean Square Deviation (RMSD) and the fraction of native contacts (fnat). For the majority of the complexes (58%), a structure of one-star or better quality was present among the 400 structures after refinement. In most of these cases, at least one of these structures was ranked in the top 100.

We found sampling, rather than scoring, to be the limiting step. In 15% of the cases, not a single one-star structure could be generated in the rigid body stage, and in an additional 22% of the cases, there were less than 10 of them among the 10 000 structures (based on RMSD alone). This leaves only 63% of the complexes for which the sampling was good. Focusing on those complexes, we found that at least a one-star was selected in all but three cases (92%), as shown in Table 3. In the majority of the cases, we found a statistically significant enrichment of one-star complexes in the top 400.

**Table 3:** Scoring of docking solutions at the rigid body stage of the CPORT docking runs.

	Top 400	Significant	Nonsignificant	Nonsignificant	Significant
		enrichment	enrichment	depletion	depletion
**One-star**	92%	63%	16%	16%	5%
**Two-star**	65%	46%	19%	35%	0%

The table indicates the percentage of cases for which at least one correct structure is selected in the top 400, and the percentage of complexes of which the number of correct structures is higher than random selection (enrichment) or lower than random selection (depletion). Significance (p<0.05) was determined using the hypergeometric distribution.

One-star and two-star criteria correspond to the CAPRI [18] definitions (see Material & Methods). For one-star structures, only those complexes are considered with at least 10 one-star solutions among the 10 000 (63% of all complexes). For two-star structures, only those complexes are considered with at least one two-star solution (44% of all complexes). The fnat criterion is not taken into account.

It was considerably more difficult to generate two-star complexes. In only 44% of all cases, a two-star complex could be generated at all during the rigid-body docking stage (Figure 1). Fortunately, the HADDOCK scoring of these solutions worked very well. In 65% of the cases where any two-star was generated, one could be selected among the top 400 (Table 3). For the large majority of those cases, this corresponds to a significant enrichment in two-star structures in the top 400. In total, after refinement, a two-star structure was present in 25% of the cases.

These successes of HADDOCK on the benchmark are largely due to the fact that the docking was driven by CPORT predictions. For comparison, we performed an *ab initio* HADDOCK docking run for every complex (Figure 1, lower graph). In only 27% of all cases, a one-star or better structure was present in the 400 refined structures in *ab initio* docking, which is less than half the success rate of CPORT. Only one complex (1IJK) was successful in *ab initio* docking but not in CPORT-driven docking (results not shown). 32% of the cases were successful for the CPORT run alone and 25% were successful for both runs. After pooling all of these categories, 71% (corresponding to 42% of all complexes) had more one-star structures in the CPORT run than in the *ab initio* run.

Two-star structures in *ab initio* docking could be generated for only 11 complexes (19%). In only four cases (7%), one or two of these two-stars were among the refined 400 structures, corresponding to less than one third of the success rate of the CPORT runs. Only in a single case (1AY7), a three-star prediction could be generated after refinement in the CPORT run (results not shown). No three-star structures were generated during *ab initio* docking.

### The effect of HADDOCK refinement

We previously found that refinement in HADDOCK does not systematically improve RMSD, but does result in significant improvements in the fraction of native contacts (fnat), if the structure is already of one-star quality [8]. This is confirmed by the current results. During the first flexible annealing refinement stage, the overall fnat increased on average by a small amount of 0.014. However, limiting the analysis to structures that are of one-star quality or better (based on RMSD alone) after the rigid body stage, the average fnat increase was 0.095. Upon water refinement, an additional gain in native contacts of 0.021 was achieved for these structures. Therefore, refinement resulted in an average gain of more than 11 percent of the native contacts. Note that this is sufficient to promote a structure without any native contacts to a one-star prediction. In terms of RMSDs, refinement had no systematic effect, with average changes of less than 0.2 Å and standard deviations of less than 1 Å for interface and ligand RMSDs.

We found refinement to have little effect on the scoring of one-star structures (results not shown). However, refinement significantly improved the scoring of two-star structures. The rank of the first two-star structure improved in 86% of the cases, and the average rank of the first two-star structure improved from 81 to 35.

### Success rate among different categories

The docking benchmark consists of different categories: enzymes (enzyme-inhibitor and enzyme-substrate complexes), antibody-antigens (not suitable for interface prediction and therefore not studied here) and other complexes. The complexes are also subdivided into rigid, medium and hard complexes, based on the conformational change upon complexation.

In general, we found large differences in success rate between the different categories. While a one-star model could be generated during refinement in 58% of the cases overall, this percentage increased to 80% for enzymes (91% of the rigid enzymes and 25% of the medium/hard enzymes) while it was only 41% for non-enzyme complexes (57% of the rigid non-enzymes and 15% of the medium/hard non-enzyme complexes). Overall, the success rate was 74% for rigid complexes and 18% for non-rigid complexes. Using interface predictions, it seems that rigid enzymes and medium/hard complexes form two extremes in docking difficulty, with rigid non-enzymes almost exactly in between.

Among all complexes, 25% contained at least one two-star structure among the 400 refined structures. All of these successful cases were rigid, forming 36% of all rigid structures. Two of them were non-enzymes, comprising 10% of all rigid non-enzymes. The remainder consisted of rigid enzymes, comprising 62% of this category.

### Docking results in relation to interface prediction results

For the 33 successful complexes (with at least one star in the top 400 refined structures), we compared the rank of the first one-star solution to the sensitivity and specificity of the interface predictions (Table S4). Overall sensitivity values were high, and no particular relationship was found between the average sensitivity and the first one-star rank (Spearman rank correlation, r = −0.28).

For specificity, however, a strong relationship was found. 14 complexes had an average specificity of more than 35%, of which 5/14 had a one-star solution at rank 1; 8/14 in the top 10; 12/14 in the top 50; and 13/14 in the top 100. In contrast, among the complexes with less than 35% average specificity, only 1/19 had a one-star solution at rank 1; 3/19 in the top 10; 6/19 in the top 50; and 10/19 in the top 100. The overall correlation between average specificity and first one-star rank was −0.53 (Spearman rank correlation).

### Post-docking interface prediction

Fernandez-Recio *et al*. [19] reversed the usual concept that interface predictions should be used in docking: analyzing the interfaces of favored *ab initio* docking solutions, they found that the interface could be predicted from these structures in most cases. Very recently, the same result was found by Hwang *et al.* [20] and also from an analysis of CAPRI predictions [21].To investigate whether this is also true for our study, we analyzed the contacts made by all 400 refined structures for every complex in the CPORT-driven run. For each of the two chains, we took the top N contact-forming residues, where N is the original number of CPORT predictions for that chain, and used these residues to “post-predict” the interface. Very difficult docking cases were excluded from the analysis, i.e. at least one good solution (one-star or better) had to be present among the top 400 predictions of the CPORT run and/or the *ab initio* run. Complexes with internal symmetry were also excluded.

We found that interface post-prediction on docking solutions can make considerable improvements on the interface predictions that drove them. In 66% of the cases, interface post-prediction improved compared to the original CPORT prediction, whereas it deteriorated in only 19%. The average sensitivity among all these complexes was 72.3%, compared to 62.3% for CPORT. This improvement in interface prediction was already apparent after rigid body docking: by analyzing the top 400 rigid body structures, average sensitivity among all complexes was 71.5%, only slightly worse than the average after refinement and much better than CPORT.

Moreover, we found that interface post-predictions could also be obtained from *ab initio* HADDOCK runs. Again, we took the top N contact-forming residues, were N was the number of CPORT predictions for that protein. After refinement, predictions were better than random for 86% of the cases, significantly so in 53% of all cases (p<0.05, hypergeometric distribution). Strikingly, in 33% of the cases, this prediction was actually better than the CPORT prediction for that chain. The average sensitivity among all analyzed complexes was 57.7%, worse than CPORT but much better than the average sensitivity of 40.7% for a random prediction. Post-prediction results were nearly identical when obtained from rigid body solutions instead of refined solutions (results not shown).

## Discussion

Here we present CPORT, a consensus docking method specifically optimized for data-driven docking in HADDOCK. Based on six interface predictors for which a web server is available, it improves upon the best-performing of those methods, PINUP. Applied to a large and diverse benchmark of complexes, CPORT interface predictions were shown to be constant and reliable, generating at least one correct prediction for all but 2% of the cases. This stable performance was confirmed on an independent test set consisting of all new complexes from benchmark 3.0. In addition, CPORT predictions were used to drive blind unbound docking using HADDOCK, resulting in an acceptable or better solution among the 400 refined structures for 58% of the complexes.

Zhou and Qin [3] found that interface predictions can be used in docking if specificity and sensitivity are both higher than 40%, limiting their use to the enzyme-inhibitor category of complexes. Here we show that interface predictions are already useful for predictions of considerable lower quality. For only 24% of the chains, the 40% sensitivity/specificity criterion was met, which means actually that for few complexes this was met for both chains. Nevertheless, we find that for 71% of the complexes, HADDOCK with CPORT interface predictions performs better than HADDOCK *ab initio* (excluding complexes that failed in both cases). For a fair comparison, it must be mentioned that Zhou and Qin used interface predictions to filter *ab initio* docking solutions, rather than using them to drive the docking.

It should be noted here that we have measured the prediction performance against the interfaces defined from the protein-protein docking benchmark. In reality, not all “false positive” predictions will be wrong: many might actually correspond to alternative interfaces (it is well known that proteins can often bind various targets). While those residues are “wrong predictions” in the context of the protein complexes defined in the benchmark, they might well be correct for interaction with other partners. Consequently, in the purpose of only predicting putative interfaces for a given protein, the reported specificities only represent lower limits, which we, however, consider to give fair measure of the performance in the context of predicting a specific complex, as is the case in this work.

The docking results obtained here are a considerable improvement over our previous efforts based on a combination of the interface predictors WHISCY and ProMate [11]. The aim of that study was merely to sample acceptable complexes (l-RMSD<10 Å) among 2000 rigid body structures, focusing on a data set of (mostly rigid) enzyme complexes. Both a meta-prediction strategy (WHISCYMATE) and a consensus strategy (Added prediction) were tried. Nevertheless, among the complexes from benchmark 2.0, the WHISCYMATE docking run generated no acceptable solutions for 6/23 cases and only one (out of 2000) in a seventh case. The Added docking run generated no acceptable solutions for 3/23 cases, only one in another 2/23 and only three in a sixth case. In contrast, in the current work, for only one of those 23 cases (1F34), no structure with l-RMSD<10 Å could be generated in the top 2000. For all other cases, at least five correct structures could be generated. Therefore, unlike the previous study, CPORT can achieve sufficient sampling for enzyme complexes in nearly all cases.

### Comparison of HADDOCK-CPORT with BDOCK-metaPPI

Huang and Schroeder [13] published a meta-predictor for protein-protein interfaces, metaPPI, designed to improve docking results, in this case in combination with their docking program BDOCK. However, their design differs in several important aspects from the present work. First, metaPPI combines interface predictors using a voting machine rather than the consensus strategy used by CPORT. Second, the predictors used by Huang and Schroeder do not include PIER and WHISCY, but do include a patch predictor, PPI-PRED [22]. The output of metaPPI is also a continuous patch, rather than a list of residues such as provided by CPORT and the other individual predictors. Finally, the predictions are used to filter the docking results, rather than to drive the docking process.

We found HADDOCK-CPORT to be superior in performance to BDOCK-metaPPI. Comparisons were made to the best performing docking method BDOCKnb, filtered by metaPPI predictions, resulting in 1500–2000 docking solutions. BDOCK-metaPPI selected at least one structure with l-RMSD<10 for 17/19 enzymes and 7/21 other complexes chosen from the benchmark 2.0 (not counting antibody-antigen complex 1KXQ). With an identical dataset, criteria and selecting the same number of structures from the rigid body stage, HADDOCK-CPORT was successful for 17/19 enzymes and 9/21 other complexes. In general, HADDOCK-CPORT generated far more acceptable structures than BDOCK-metaPPI: using a more strict criterion of at least four acceptable structures in the selected 1500–2000 structures, the success rate for BDOCK-metaPPI dropped to 15/19 and 5/21 for enzymes and other complexes, respectively, but the success rate for HADDOCK-CPORT remained 17/19 for enzymes and became 7/21 for other complexes.

### Comparison of HADDOCK-CPORT with the SVM method of Martin and Schomburg

Martin and Schomburg [23] trained a Support Vector Machine (SVM) method to score docking solutions, exploiting several properties also used in interface prediction, such as interface propensity and conservation, as well as other properties. Unlike the simple optimization scheme used by CPORT, machine learning methods such as SVMs contain hundreds of parameters that are optimized, and therefore great care must be taken to prevent over-fitting.

The SVM program was trained separately on docking solutions from each of the three classes from the benchmark (enzyme, antibody-antigen and other) and tested on different docking solutions from the same complexes. Therefore, the program was implicitly aware on the characteristics of correct solutions for each of the complexes in the benchmark. In contrast, the current work is a blind docking study: neither HADDOCK nor CPORT was aware of the correctness or incorrectness of any docking solution of the complexes during prediction, docking or scoring.

Nevertheless, we found HADDOCK-CPORT to achieve the same performance as the SVM method of Martin and Schomburg. As a criterion for success, Martin and Schomburg used the presence of at least one structure of i-RMSD<5 Å among the top 100 structures, which they achieved for 26/51 complexes. For HADDOCK-CPORT, this was achieved for 29/59 complexes. Comparison on the class level is not possible since Martin and Schomburg classified some complexes differently than currently annotated in the benchmark.

### Comparison of HADDOCK-CPORT with ZDOCK-ZRANK

ZDOCK [24], [25] is an FFT-based *ab initio* rigid body docking method, one of the top performing methods together with HADDOCK in the CAPRI experiment [26], [27]. The protein-protein docking benchmark has been developed by the ZDOCK group, who has used it in the optimization of docking and scoring methodology. It serves as an important testing ground for new developments in docking methodology.

A scoring function called ZRANK has been developed by the ZDOCK group, with the goal to re-rank ZDOCK solutions [28]. Using ZRANK and version 3.0 of ZDOCK, Pierce and Weng [29] managed to score a hit (structure with i-RMSD<2.5 Å) among the top 100 in more than 50% of the complexes in the benchmark 2.0, while 60% contains a near-hit (structure with i-RMSD<4.0 Å). This sets a formidable standard for any docking method.

It is not the goal of the present study to outperform *ab initio* docking methods in the context of docking with zero experimental data. Unlike HADDOCK, ZDOCK is optimized for such cases, containing features such as shape complementarity [30], [31] and statistical contact potentials [32] that are absent in HADDOCK. Rather, the strength of HADDOCK lies in its flexible use of experimental data. The present study aims to establish a prediction-driven docking protocol that experimentalists can use as a starting point, incorporating experimental data and expert knowledge in the form of filtering the predictions, adding additional constraints, cutting up the protein or any of the myriads of other possibilities offered by HADDOCK, and that will directly improve the results.

Still, we compared the *ab initio* results of ZDOCK-ZRANK to the blind data-driven docking results of HADDOCK-CPORT. The ZDOCK-ZRANK supplementary material was downloaded and analyzed, limiting the analysis to the same set of complexes used here. Since the supplementary material contains only i-RMSD values, and no l-RMSD or fnat values, a comparison based on CAPRI criteria is not possible. Therefore, we used the near-hit definition (i-RMSD<4.0 Å) as criterion of success, since it is similar to the CAPRI one-star criterion.

We found HADDOCK-CPORT to be competitive with ZDOCK-ZRANK in terms of near-hits (Table 4). Among the top 400, HADDOCK-CPORT selected a near-hit in 35/59 cases, the same performance as ZDOCK-ZRANK. However, in three cases, the near-hit deteriorated and was lost during refinement. In addition, ZDOCK-ZRANK was also better able to rank near-hits in the top 100.

**Table 4:** Comparison between HADDOCK-CPORT and ZDOCK-ZRANK.

	Top 10 000	Top 400	Top 100
***i-RMSD<4.0***			
ZDOCK-ZRANK	52	35	25
HADDOCK-CPORT,			
rigid body stage	45	35	21
HADDOCK-CPORT,			
refinement stage	-	32	21
***i-RMSD<2.5***			
ZDOCK-ZRANK	38	25	21
HADDOCK-CPORT,			
rigid body stage	26	18	9
HADDOCK-CPORT,			
refinement stage	-	19	13

Success rate of HADDOCK-CPORT compared to ZDOCK-ZRANK among 59 “enzyme” and “other” complexes from the protein-protein docking benchmark. Shown is the number of successful complexes for each method.

Although not corresponding to any CAPRI criterion, we also looked at the generation and scoring of what Pierce and Weng defined as “hits” (i-RMSD<2.5). Here we found that HADDOCK-CPORT was outperformed by ZDOCK-ZRANK. After the rigid body stage, in 18/59 cases at least one hit scored among the top 400 for HADDOCK-CPORT. During refinement, this increased to 19/59, which is still significantly less than the 25/59 complexes that were successful using ZDOCK-ZRANK. The complexes that yielded hits in the top 400 with HADDOCK-CPORT usually also did so with ZDOCK-ZRANK. However, there were two complexes (1GP2 and 2MTA) that were successful for HADDOCK-CPORT and not for ZDOCK-ZRANK.

The properties used in PINUP were used by the authors to enrich the number of hits generated by ZDOCK [33]. They achieved a six-fold enrichment over the native ZDOCK score, leading to a performance comparable to ZRANK.

### Conclusions

Protein-protein docking can be and has been applied to a wide range of complexes, ranging from cases where extensive experimental information about the interface is available, to cases where docking is completely blind, i.e. no other information is known than that the proteins do interact. The large majority of the docking methods are *ab initio* methods, designed towards the latter class of complexes, although experimental data can often be incorporated to restrict the search space. In contrast, HADDOCK is a data-driven docking program that has been widely used in combination with experimental data, ranging from NMR data, mass spectrometry data to mutagenesis data (for a comprehensive overview, see van Dijk *et al.* [9]). *Ab initio* docking in HADDOCK, while possible, performs poorly compared to state-of-the-art docking methods limiting HADDOCK to cases where sufficient experimental data is available. While these data are much easier to obtain than the actual experimental structure of the protein complex, this has been an important limitation of the data-driven docking paradigm compared to *ab initio* methods.

Here we have demonstrated that this limitation is removed when data-driven docking is combined with consensus interface predictions. While interface predictions have been used previously in docking, their success has been mostly limited to cases for which interface prediction is relatively easy, such as rigid enzymes [11] or enzyme-inhibitors [3] In the current work, by using a consensus prediction strategy in combination with improved docking protocols, much improvement has been made over earlier attempts.

We have demonstrated that sampling, rather than scoring, is the limiting step when using interface predictions in HADDOCK. Even if only 0.1% (10/10 000) of the sampled structures is of one-star quality or better, one can be selected among the top 400 in basically all cases. For two-star structures, the sampling of even a single structure is often enough. We have also shown that flexible refinement is helpful in improving the fraction of native contacts in the docking models, and in improving the rank of two-star structures among the selected structures.

The current study shows that using interface predictions, the performance gap between data-driven docking and *ab initio* docking methods for blind docking cases is nearly closed. It is not the purpose of this study to develop a docking protocol to replace *ab initio* docking. Indeed, our results show that in the complete absence of experimental information, ZDOCK-ZRANK is still somewhat better than HADDOCK-CPORT. However, the small difference in performance makes HADDOCK-CPORT an excellent starting point for cases with limited experimental data. Blind docking driven by interface predictions represents the absolute bottom line of what is possible in HADDOCK: since HADDOCK is designed to incorporate experimental data in the most powerful and flexible way, any additional biological knowledge will greatly improve the results obtained by prediction-driven docking alone.

In this light, it is also encouraging that the initial estimate of the interface given by consensus interface prediction can also be improved by post-prediction based on the top-scoring docking results. In data-driven docking, these new predictions can be used directly in a new docking run. Used together with expert knowledge in the interpretation of interface predictions and docking results, prediction-driven docking is a powerful new tool in the generation of new hypotheses on the atomic details of macromolecular interactions.

All docking structures described in this work represent a very extensive decoy set which is freely available for download at http://haddock.chem.uu.nl/services/CPORT/cport-suppmat.html. They contain both high-quality refined structures and energy-minimized rigid-body structures. We hope that this decoy set will be useful for the ongoing development of new scoring methods. The decoy set also contains a large number of additional statistics regarding the performance of the various interface predictors and the different docking stages. These include, among other, the sensitivity and specificity of each interface predictor for both chains, the number of one-star, two-star and three-star solutions at each of the docking stages, and also their ranks.

A web server for CPORT has been developed using the Spyder framework for data-driven programming [34]. The CPORT web server is freely available at http://haddock.chem.uu.nl/services/CPORT.

Finally, the optimized protocol for prediction-driven docking has been made available as a special web server interface in the HADDOCK web server, and can be accessed at http://haddock.chem.uu.nl/services/HADDOCK/haddockserver-prediction.html.

## Materials and Methods

### Dataset

Prediction and docking were performed on the full docking benchmark 2.0 [5], with the exception of antibody-antigen complexes and homotrimeric/homotetrameric complexes (see below). Complexes classified as “medium” and “hard” were included. We followed the re-classification of 1FQ1, 1IJK and 1M10 as “enzyme” in the recent version of the benchmark [17].

### Quaternary state

In some of the complexes, one partner is a symmetric homodimer, homotrimer or homotetramer. The interface between the subunits is usually obligate and shows a strong signal in interface prediction. Therefore, in the case of homodimers (1A2K, 1AKJ, 1EER, 1IB1, 1ML0) predictions and docking were performed on the dimer rather than the monomer, to prevent the dimer interface from being predicted. Predictions were not forced to be consistent between subunits. In the RMSD evaluation of docking solutions (see below), each dimer was fitted and evaluated onto the reference structure in both possible ways, and the best of the two statistics was taken.

This procedure was considered too complex for the three complexes with homotrimers/homotetramers (1KKL, 1RLB and 1N2C), and therefore, these complexes were excluded beforehand.

### Interface prediction

Predictions were retrieved from the web servers of WHISCY [11], PIER [14], ProMate [35], cons-PPISP [36], SPPIDER [37], and PINUP [38], using default settings and unbound structures. Due to technical problems with the PINUP server, some of the PINUP predictions were run locally using source code and binaries kindly provided by the authors. For all predictors, the prediction scores were used as returned by the web server. In case of cons-PPISP, which returns a set of clusters, the predictions were converted to a score based on the cluster rank, rather than the confidence score provided for each cluster (see Text S1). The score was computed as 100 * c+n−1, where c is the rank of the predicted cluster to which the residue belongs, and n the rank of that residue within the cluster. Residues not belonging to any cluster were given a score of 10 000. Residues predicted to be buried were given a score of 100 000.

### Integrating interface prediction scores

A consensus prediction method was developed on a subset of the residues in the benchmark complexes (the “training set”). Every residue was classified as interface or non-interface; residues were considered to be interface residues if the shortest heavy-atom distance to the partner protein was less than 5 Å. All residues and chains for which one or more predictors gave no result were discarded, which includes all interface and non-interface residues that one or more predictors classified as buried. This resulted in a training set of 14480 residues, of which 2243 (15.1%) were interface residues. PIER predictions where filtered according to surface accessibility, considering only residues with a relative side chain or main chain accessibility of at least 15% as determined by NACCESS [39].

Then, for PIER, WHISCY, ProMate and PINUP, for every chain the scores were ranked and the ranks were written in the table. For cons-PPISP, all scores were pooled, sorted from low to high, and divided into N partitions, where N is the average number of predictable residues per chain. For every score, the partition to which it belonged was determined and this was written as rank into the table. The same procedure was applied to SPPIDER scores, except that these were sorted from high to low. All ranks were capped at 30.

For the integration of scores, a consensus strategy was chosen, adding predictions rather than combining them into a new score. For every predictor, a threshold variable was defined. A residue with rank in any predictor lower or equal than the threshold of that predictor was considered to be selected. To choose the optimal set of thresholds, a simple, greedy algorithm was used. All thresholds were initialized to zero, starting with an empty set of predictions. Then, for every interface predictor, a threshold increment of one was tried; then, out of the six resulting threshold sets, the set was chosen that resulted in the largest specificity. This procedure was repeated until all residues were predicted. This resulted in not a single threshold set, but a list of them, each corresponding to the optimal prediction set for a given cutoff.

### Consensus interface prediction in docking

Consensus predictions were made at three different cutoffs, corresponding to a balanced prediction, slight overprediction and heavy overprediction, respectively:

- The first cutoff (balanced prediction) corresponds to taking the top 4 WHISCY predictions, the top 3 PIER predictions, the top 6 ProMate predictions and the top 12 PINUP predictions, no cons-PPISP predictions, and the top 6 score partitions (scores higher than 91.02) of SPPIDER, resulting on average in 22 predictions per chain.
- The second cutoff (slight overprediction) corresponds to taking the top 6 WHISCY predictions, the top 7 PIER predictions, the top 11 ProMate predictions and the top 13 PINUP predictions, the 11 highest score partitions (corresponding with the top 14 of cluster 1) of cons-PPISP, and the top 6 score partitions (scores higher than 91.02) of SPPIDER, resulting on average in 33 predictions per chain.
- The third cutoff (heavy overprediction) corresponds to taking the top 14 WHISCY predictions, the top 20 PIER predictions, the top 19 ProMate predictions and the top 21 PINUP predictions, the 14 highest score partitions (corresponding with the top 14 of cluster 1) of cons-PPISP, and the top 6 score partitions (scores higher than 91.02) of SPPIDER, resulting on average in 50 predictions per chain.

Initial docking tests showed the third cutoff to be optimal in docking (see Text S1), and therefore, this cutoff was chosen for the CPORT consensus predictor. In cases where a predictor was unable to yield a prediction on a particular chain, consensus predictions were made using the consensus of the remaining interface predictors.

### Evaluation of interface predictions

Residues were considered to be interface residues if the shortest heavy-atom distance to the partner protein was less than 5 Å. We defined surface residues in the same manner as in the WHISCY paper [11], as residues with a relative side chain or main chain accessibility of at least 15% as determined by NACCESS [39]. This resulted in 14% of the surface residues to be defined as interface. This resulted in an “evaluation set” of 20185 residues, of which 2987 (14.8%) were interface residues.

Interface predictions were classified as true positives (TP), false positives (FP), true negatives (TN) and false negatives (FN), and evaluated using the following criteria:

- *Sensitivity*, corresponding to the fraction of the interface that was successfully predicted, defined as TP/(TP+FN).
- *Specificity*, corresponding to the fraction of the predictions that were correct, defined as TP/(TP+FP).

The evaluation set is overlapping, but not identical, to the training set that was used to develop the CPORT thresholds and cutoffs. All of the training set residues were in the test set, but they formed only 71.7% of the evaluation set. This has several causes. First, the number of chains in the evaluation set was somewhat larger, since for some interface predictors, predictions could be obtained after some re-formatting of the structure, which was done after development. This final set of chains (109) consisted of all chains in the benchmark except 1FC2 chain C, 1ML0 chain A, 1PPE chain I and both chains of 1HE8, 2PCC and 1H1V.

Second, unlike the development set, the evaluation set of predictions contained all residues that we defined as surface residues, regardless of whether they received a score from all predictors. Finally, all interface residues were considered in the evaluation, regardless of whether they received a score from all predictors or whether they passed the surface accessibility criterion. In fact, out of the 2987 true interface residues, 423 (14.2%) were missing or buried in the unbound structure. This means that only 13.0% of the evaluation set consisted of interface residues that could possibly be predicted, and this would be the expected accuracy of a random predictor. In addition, this meant that the maximum achievable sensitivity was only 85.8%.

To determine the relative performance of the six individual predictors, the top 30 predictions of every method were taken.

An independent evaluation was performed on all 74 new chains of benchmark 3.0 [17] that are not antibody-antigens. It was verified that these chains form a validation set that is largely orthogonal in sequence homology,: of the new chains, only 7 are also present in the benchmark 2 (with completely different partners), and an additional 8 have an homologue. That leaves 59 new chains with less than 30% sequence identity to any chain in the benchmark 2.

For the benchmark 2.0, to compare the best predictor, PINUP, with CPORT, the top 50 PINUP predictions were taken so that the number of predictions was on average equal between the two methods. For the benchmark 3.0, where CPORT made fewer predictions (45 on average), the top 45 PINUP predictions were taken for comparison, to make sure that an equal number of predictions was made by both PINUP and CPORT.

### Prediction-driven docking

CPORT prediction-driven docking was performed using HADDOCK 2.1 and the “haddock” and “haddockserver” modules of the Spyder framework, which are part of the HADDOCK server [34]. For chains where not all predictors gave a result, the CPORT consensus of the remaining predictors was used. Docking was performed with the following parameter settings: 10 000 structures in the rigid body stage, ntrials = 1, noecvpart = 8/7, meaning that for every structure, 87.5% of the restraints were discarded at random. An alternative CPORT run was performed with 5000 rigid body structures, ntrials = 5 (for every structure, rigid-body docking was performed 5 times with the best structure written to disk).

Predictions were translated into ambiguous interaction restraints (AIRs) in the standard way, defining predicted residues as active residues and surrounding surface residues (within 6.5 Å from any active residue) as passive residues. For each active residue, HADDOCK defines an AIR restraint between that residue and all active and passive residues of the partner protein.

### *Ab initio* docking

HADDOCK *ab initio* docking was performed using center-of-mass restraints: during the initial rigid body minimization, a distance restraint was defined between the centers-of-mass of the respective partners (this was done by defining a distance restraint between the CA atoms of each protein with the center averaging option in CNS [40]); the upper distance limit was automatically defined from the dimensions of each protein along the x,y,z axis of the molecular alignment tensor as: d_center-of-mass_ = 1+(dx_i_+dy_i_+dz_i_+dx_j_+dy_j_+dz_j_)/12 were i and j indicates the two proteins, respectively. 10 000 rigid body structures were generated with ntrials = 5.

### Refinement of docking solutions

For all docking runs, the top 400 structures after rigid body docking were selected for the subsequent two refinement stages: flexible simulated annealing in torsion angle space (it1) and flexible water refinement in Cartesian space (water). To save computation time, the refinement stage was only run for only those complexes with at least one one-star (not taking into account fnat) in the rigid body stage of either the CPORT run or the *ab initio* run (40 out of 59 complexes). If this criterion was met for only one run, both runs were nevertheless refined. The complexes for which this criterion was not met for either run were considered failures and were not refined.

The docking calculations were performed on the Mare Nostrum Supercomputer, Barcelona, Spain, and on the HADDOCK server cluster in Utrecht. Each run (with refinement) required around a total of 200 CPU hours.

### Evaluation of docking solutions

Stars were awarded according to CAPRI criteria [18]. For a complex to be classified as one-star, its interface root mean square deviation (i-RMSD) from the complex has to be lower than 4 Å or its ligand RMSD (l-RMSD) lower than 10 Å. In addition, the fraction of native contacts (fnat) has to be > = 0.1. For two-star predictions, the criteria are i-RMSD<2 or l-RMSD<5, and fnat> = 0.3. For three-star predictions, the criteria are i-RMSD<1 or l-RMSD<1, and fnat > = 0.5. In evaluating the rigid body stage of docking, the fnat criterion was not applied, because a significant improvement in fnat is usually observed upon HADDOCK flexible refinement. In evaluating symmetry-related complexes (1A2K, 1AKJ, 1EER, 1F51, 1IB1, 1ML0), the fnat criterion was never applied.

### Interface post-prediction

Interface post-predictions were made on all docking runs (CPORT-driven and ab-initio) that were subjected to refinement (see above), except for complexes with internal symmetry. For each chain, predictions were made by ranking the residues according to the number of contacts made by that residue among all selected docking solutions. For post-predictions from water-refined structures, all structures were selected, whereas only the top 400 structures were selected for predictions from rigid body structures.

### Availability and Requirements

The CPORT web server if freely available for use without any restriction and registration requirements from the following web address: http://haddock.chem.uu.nl/services/CPORT.

## Supporting Information

Figure S1 **Optimizing the best use of each individual predictor.**
(PDF)

Figure S2 **Predictions on the bound form (black) versus the unbound form (dashed red).**
(PDF)

Table S1 **Comparison between CPORT and other predictors.** Comparison between CPORT and the top 50 predictions of PINUP, WHISCY, PIER, ProMate, SPPIDER and cons-PPISP on the benchmark 2.0.
(PDF)

Table S2 **Comparison between CPORT and other predictors.** Comparison between CPORT, the top 50 PINUP predictions (PINUP) and a simple meta-predictor (meta-2 and meta-3; selects residues that are in the top 30 of two or more or more/three or more interface predictors) on the benchmark 2.0. On the benchmark 2.0, CPORT made on average 50 predictions per chain.
(PDF)

Table S3 **Comparison between CPORT and other predictors on the benchmark 3.0.** Comparison between CPORT, the top 45 PINUP predictions (PINUP) and a simple meta-predictor (meta-2 and meta-3; selects residues that are in the top 30 of two or more or more/three or more interface predictors) on the 37 new targets of the benchmark 3.0. On these targets, CPORT made on average 45 predictions per chain.
(PDF)

Table S4 **CPORT sensitivity and specificity versus rank of the first one-star structure.**
(PDF)

Text S1 **Description of the optimization of docking parameters.**
(PDF)
