# Supplementary material for: CPORT: A Consensus Interface Predictor and Its Performance in Prediction-Driven Docking with HADDOCK
Source: PLoS One. 2011 Mar 25;6(3):e17695. doi: 10.1371/journal.pone.0017695 (PMC3064578; doi:10.1371/journal.pone.0017695)
Supplement: Figure S1 — Optimizing the best use of each individual predictor. (PDF) [file pone.0017695.s001.pdf]

Figure S1: Optimizing the best use of each individual predictor

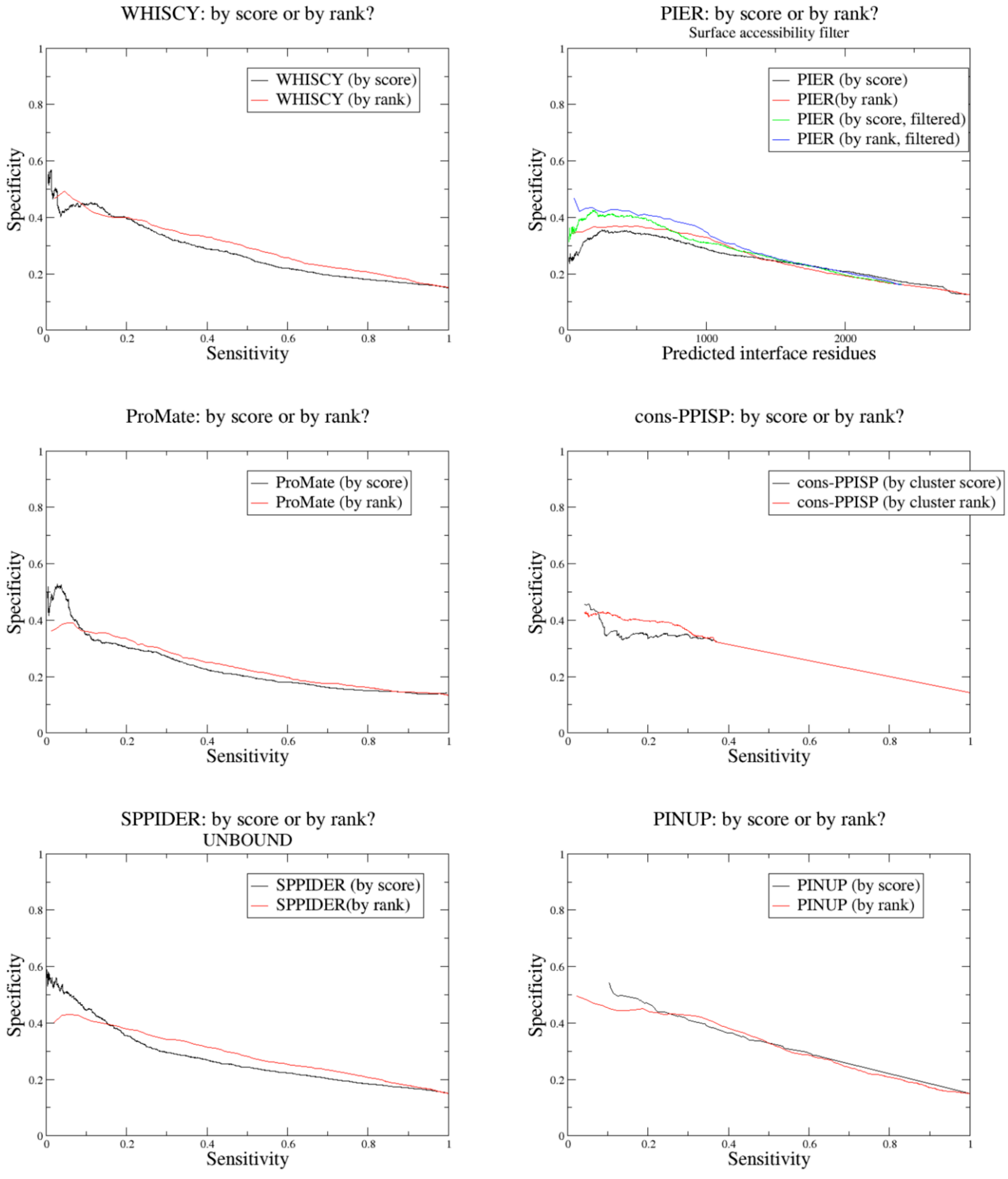

Before the scores were combined, we investigated what would be the optimal way to use the scores provided by each predictor. In our previous work, we selected all WHISCY scores higher than 0.18 as predictions in HADDOCK, rather than taking the top-ranking predictions. However, to our surprise, we found that WHISCY predictions have a higher specificity when the top ranking predictions are taken, rather than using an absolute score cutoff, especially when higher sensitivities are desired (figure S1A). The same applies to PINUP predictions (S1F), although the differences are very small in this case.

For PIER, we performed the same analysis. However, we noticed that many more residues were given a score than in WHISCY or PINUP. This is due to the fact that the surface accessibility cutoff, the value above which a residue is considered a surface residue and hence a potential interface residue, is quite liberal in PIER. Therefore, we filtered PIER predictions by eliminating all predictions that did not pass the surface accessibility criterion in WHISCY, i.e. relative surface accessibility of at least 15 % for either main chain or side chain. As evident from figure S1B, this led to a substantial increase in the specificity of PIER. As in WHISCY and PINUP, PIER predictions performed best if the top-ranking residues were taken, rather than all residues with a score above a certain threshold.

Cons-PPISP is a neural network method that returns predicted interface clusters, rather than scores for every residue. However, a confidence score is given to every cluster. We tested if cons-PPISP gives better result if the confidence score of the cluster is used or whether a score based on the rank of a clusters and of the residue within the cluster is used instead; we found the latter to be the case (figure S1D).

ProMate was designed for high specificity, rather than high sensitivity. This is reflected in figure S1C, showing that very high ProMate scores are a better predictor than high ProMate ranks. However, since our goal is high sensitivity, we used ProMate ranks instead of scores, since ranks perform better at higher sensitivity. In general, we found that ranks are less sensitive to conformational changes (i.e. making predictions on the bound or unbound form; results not shown), so we chose ranks over scores unless we had a good reason to do otherwise.

For SPPIDER, however, we found SPPIDER values of all methods to have the highest specificity at high scores (figure S1E). Because of this, and because the residue properties used in SPPIDER are rather special (predicted minus observed surface accessibility), we considered it better for the orthogonality of the predictions to use SPPIDER scores, rather than SPPIDER ranks, even though SPPIDER ranks perform better at high desired sensitivities.
