# Supplementary figures and images for: CPORT: A Consensus Interface Predictor and Its Performance in Prediction-Driven Docking with HADDOCK
Source: PLoS One. 2011 Mar 25;6(3):e17695. doi: 10.1371/journal.pone.0017695 (PMC3064578; doi:10.1371/journal.pone.0017695)

**Figure S2: Predictions on the bound form (black) versus the unbound form (dashed red)**

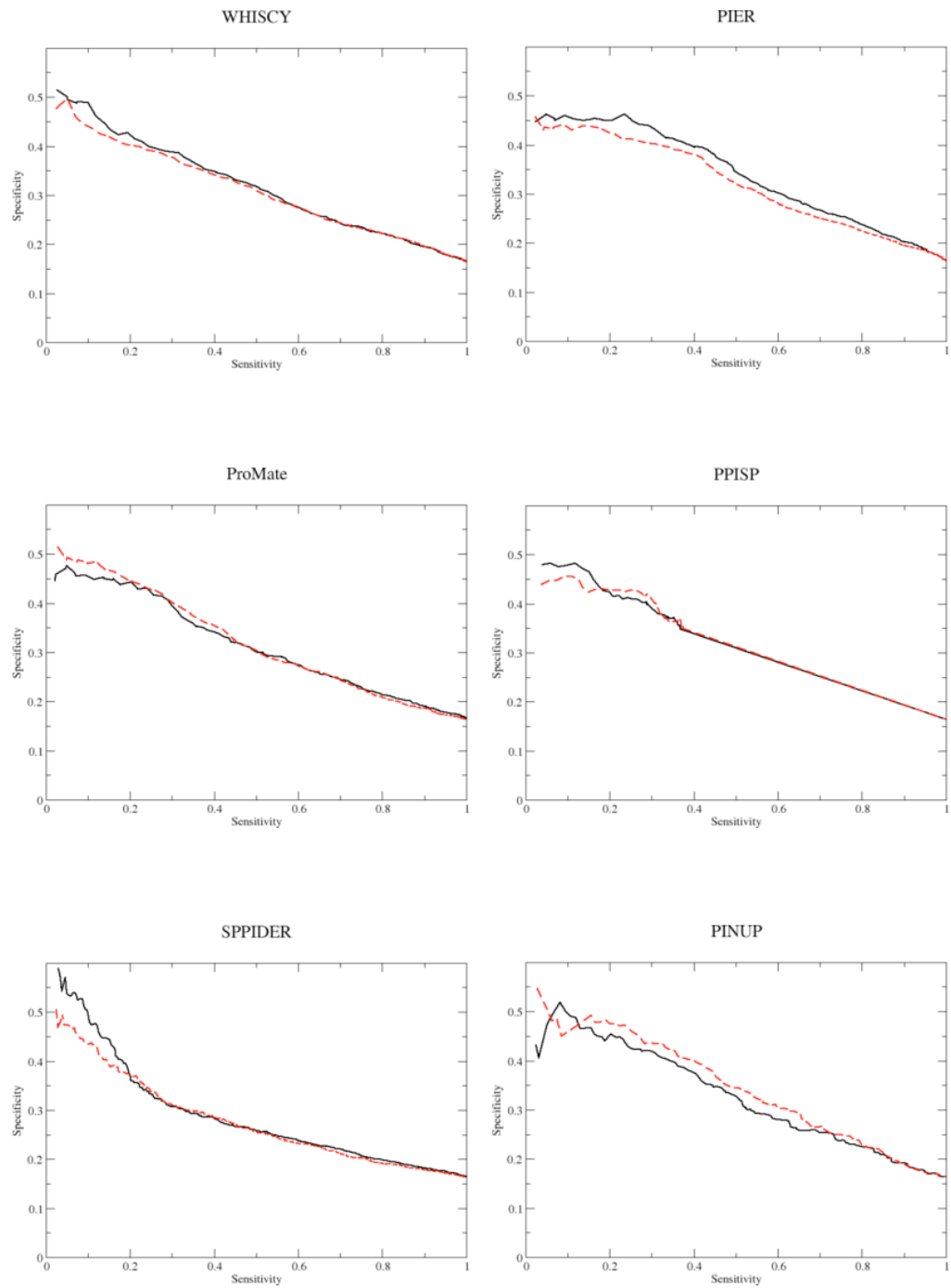

Supplement: Figure S2 — Predictions on the bound form (black) versus the unbound form (dashed red). (PDF) [file pone.0017695.s002.pdf]
