# Supplementary material for: CPORT: A Consensus Interface Predictor and Its Performance in Prediction-Driven Docking with HADDOCK
Source: PLoS One. 2011 Mar 25;6(3):e17695. doi: 10.1371/journal.pone.0017695 (PMC3064578; doi:10.1371/journal.pone.0017695)
Supplement: Table S1 — Comparison between CPORT and other predictors. Comparison between CPORT and the top 50 predictions of PINUP, WHISCY, PIER, ProMate, SPPIDER and cons-PPISP on the benchmark 2.0. (PDF) [file pone.0017695.s003.pdf]

Table S1 - Comparison between CPORT and other predictors.

Comparison between CPORT and the top 50 predictions of PINUP, WHISCY, PIER, ProMate, SPPIDER and cons-PPISP on the benchmark 2.0.

	**All wrong**	**Sensitivity >= 40 %**	**Specificity >= 40 %**	**Sens & spec >= 40 %**	**Overall sensitivity**	**Overall specificity**
**CPORT**	2 %	82 %	24 %	24 %	53 %	27 %
**PINUP**	4 %	80 %	19 %	19 %	52 %	27 %
**WHISCY**	6 %	69 %	12 %	12 %	46 %	24 %
**PIER**	5 %	69 %	14 %	14 %	49 %	25 %
**ProMate**	3 %	65 %	17 %	17 %	47 %	24 %
**SPPIDER**	1 %	62 %	7 %	7 %	45 %	23 %
**cons-PPISP**	9 %	58 %	10 %	10 %	41 %	21 %
