# Supplementary material for: CPORT: A Consensus Interface Predictor and Its Performance in Prediction-Driven Docking with HADDOCK
Source: PLoS One. 2011 Mar 25;6(3):e17695. doi: 10.1371/journal.pone.0017695 (PMC3064578; doi:10.1371/journal.pone.0017695)
Supplement: Table S2 — Comparison between CPORT and other predictors. Comparison between CPORT, the top 50 PINUP predictions (PINUP) and a simple meta-predictor (meta-2 and meta-3; selects residues that are in the top 30 of two or more or more/three or more interface predictors) on the benchmark 2.0. On the benchmark 2.0, CPORT made on average 50 predictions per chain. (PDF) [file pone.0017695.s004.pdf]

Table S2 - Comparison between CPORT and other predictors.

Comparison between CPORT, the top 50 PINUP predictions (PINUP) and a simple meta-predictor (meta-2 and meta-3; selects residues that are in the top 30 of two or more or more / three or more interface predictors) on the benchmark 2.0. On the benchmark 2.0, CPORT made on average 50 predictions per chain.

	**All wrong**	**Sensitivity >= 40 %**	**Specificity >= 40 %**	**Sens & spec >= 40 %**	**Overall sensitivity**	**Overall specificity**
**CPORT**	2 %	82 %	24 %	24 %	53 %	27 %
**PINUP**	4 %	80 %	19 %	19 %	52 %	27 %
**Meta-2**	1 %	93 %	2 %	2 %	67 %	21 %
**Meta-3**	5 %	72 %	26 %	23 %	49 %	29 %
