# Supplementary material for: CPORT: A Consensus Interface Predictor and Its Performance in Prediction-Driven Docking with HADDOCK
Source: PLoS One. 2011 Mar 25;6(3):e17695. doi: 10.1371/journal.pone.0017695 (PMC3064578; doi:10.1371/journal.pone.0017695)
Supplement: Table S3 — Comparison between CPORT and other predictors on the benchmark 3.0. Comparison between CPORT, the top 45 PINUP predictions (PINUP) and a simple meta-predictor (meta-2 and meta-3; selects residues that are in the top 30 of two or more or more/three or more interface predictors) on the 37 new targets of the benchmark 3.0. On these targets, CPORT made on average 45 predictions per chain. (PDF) [file pone.0017695.s005.pdf]

Table S3 - Comparison between CPORT and other predictors on the benchmark 3.0.

Comparison between CPORT, the top 45 PINUP predictions (PINUP) and a simple meta-predictor (meta-2 and meta-3; selects residues that are in the top 30 of two or more or more / three or more interface predictors) on the 37 new targets of the benchmark 3.0. On these targets, CPORT made on average 45 predictions per chain.

	**All wrong**	**Sensitivity >= 40 %**	**Specificity >= 40 %**	**Sens & spec >= 40 %**	**Overall sensitivity**	**Overall specificity**
**CPORT**	3 %	70 %	26 %	24 %	48 %	28 %
**PINUP**	16 %	54 %	19 %	19 %	42 %	25 %
**Meta-2**	4 %	68 %	38 %	36 %	51 %	33 %
