# Supplementary material for: CPORT: A Consensus Interface Predictor and Its Performance in Prediction-Driven Docking with HADDOCK
Source: PLoS One. 2011 Mar 25;6(3):e17695. doi: 10.1371/journal.pone.0017695 (PMC3064578; doi:10.1371/journal.pone.0017695)
Supplement: Table S4 — CPORT sensitivity and specificity versus rank of the first one-star structure. (PDF) [file pone.0017695.s006.pdf]

Table S4 - CPORT sensitivity and specificity versus rank of the first one-star structure.

**Complex**	**CPORT predictions**				**Rank of the first one-star structure**	**# of one-star structures**
	**Receptor**		**Ligand**			
	**Sensitivity**	**Specificity**	**Sensitivity**	**Specificity**		
1ACB	0.769	0.541	1.000	0.357	29	16
1AK4	0.800	0.327	1.000	0.175	205	5
1AKJ	0.346	0.136	0.485	0.239	47	6
1AVX	0.576	0.345	0.933	0.241	113	5
1AY7	0.750	0.218	0.882	0.500	5	15
1B6C	0.571	0.179	0.591	0.325	1	8
1BUH	0.500	0.145	0.722	0.342	226	3
1BVN	0.606	0.339	0.905	0.452	1	*48*
1CGI	0.543	0.487	0.905	0.594	20	*53*
1DFJ	0.615	0.511	0.576	0.373	1	*50*
1E6E	0.483	0.292	0.724	0.600	1	*49*
1E96	0.625	0.208	0.588	0.286	50	*24*
1EAW	0.600	0.429	0.938	0.441	8	*10*
1EWY	0.381	0.190	0.778	0.452	10	*18*
1EZU	0.645	0.328	0.535	0.500	365	*1*
1F51	0.469	0.238	0.704	0.432	119	*9*
1FC2	0.529	0.127	0.733	0.314	349	*1*
1GP2	0.600	0.467	0.452	0.215	107	*9*
1HE1	0.818	0.500	0.656	0.375	2	*10*
1HIA	0.379	0.212	1.000	0.417	83	*31*
1I4D	0.385	0.139	0.526	0.294	109	*3*
1MAH	0.633	0.339	0.636	0.341	59	*3*
1ML0	0.207	0.125	0.680	0.472	62	*4*
1PPE	0.441	0.357	1.000	0.517	1	*86*
1TMQ	0.600	0.356	0.767	0.535	1	*70*

1UDI	0.630	0.354	0.759	0.550	37	*3*
1WQ1	0.459	0.362	0.724	0.512	47	*6*
2BTF	0.500	0.182	0.621	0.333	234	*1*
2MTA	0.565	0.220	0.938	0.385	72	*18*
2PCC	0.400	0.140	0.438	0.212	244	*1*
2SIC	0.645	0.400	1.000	0.271	4	*45*
2SNI	0.581	0.360	1.000	0.593	78	*12*
7CEI	0.500	0.173	0.750	0.319	31	*5*
