# Supplementary material for: CPORT: A Consensus Interface Predictor and Its Performance in Prediction-Driven Docking with HADDOCK
Source: PLoS One. 2011 Mar 25;6(3):e17695. doi: 10.1371/journal.pone.0017695 (PMC3064578; doi:10.1371/journal.pone.0017695)
Supplement: Text S1 — Description of the optimization of docking parameters. (PDF) [file pone.0017695.s007.pdf]

Optimization of docking parameters

We chose to evaluate CPORT predictions for three possible cutoffs, corresponding to a balanced prediction (a number of predictions equal to the size of the average interface), slight overprediction and heavy overprediction, respectively. For the first cutoff, sensitivity and specificity were both 39 % for the working set. This corresponded to predicting 14 % of the surface as interface, making on average 22 predictions per chain. The second cutoff, corresponding to slight overprediction, was set to 50 % sensitivity, resulting in 34 % specificity. This corresponded to predicting 20 % of the surface, on average 33 predictions per chain. The third cutoff predicted as much of 30 % of the surface as interface, making on average 50 predictions per chain. Sensitivity and specificity were at 53 % and 27 %, respectively.

We decided to determine the optimal prediction cutoff for data-driven docking. For this, each of the three CPORT prediction cutoffs was tested. Six protein complexes were chosen to optimize the docking performance: 1ACB, 1BUH, 1EER, 1EWY, 1GRN and 1KXP. These complexes are representative for the benchmark as a whole in terms of interface prediction performance, conformational change and enzyme/non-enzyme classification.
Docking was performed by generating 10 000 rigid body structures. Optimization was only performed in terms of the number of one-star or better structures generated in the rigid body stage: no scoring or refinement was performed.

In HADDOCK, ambiguous data such as interface predictions are translated into ambiguous interaction restraints. An important parameter is the number of partitions into which the data are randomly divided (*noecvpart*): for each docking trial, only one partition is used to drive the docking process. In previous versions, *noecvpart* was limited to integer values, resulting in maximum 50 % of the restraints to be discarded. In the server version of HADDOCK, it is now possible to set *noecvpart* to non-integer values, which allows discarding any desired fraction of the restraints. The following percentages of discarded restraints were tested: 50 %, 65 %, 75 %, 85 %, 90 % and 93 %. This parameter and the CPORT cutoff were the only parameters that were optimized.

To our surprise, docking results were best when very large percentages of the restraints were discarded, together with the highest CPORT cutoff. Discarding 85 % or 90 % yielded the best results, and only slightly superior to discarding 93 %, but much better than discarding fewer restraints. We took the average of 85 % and 90 % (87.5 % of the restraints discarded, corresponding with a *noecvpart* value of 8/7) as the optimal value for CPORT-driven docking with HADDOCK.
